# In conversation with…Jim Sibree Milledge

**DOI:** 10.1186/s13728-015-0030-0

**Published:** 2015-08-22

**Authors:** Ned Gilbert-Kawai

**Affiliations:** University College London Centre of Altitude, Space and Extreme Environment (CASE) Medicine, London, UK

## Abstract

*Whilst attending a conference last year, I was fortunate enough to be seated at lunch next to a very eminent octogenarian academic. Conversation ensued, and I was instantly captivated by his stories. These were not narratives filled with scientific facts but rather anecdotes of his life and the pathways he had taken to get where he was today. Fascinated by such accounts, I set about the task of interviewing persons of scientific acclaim, to learn more about their life stories and unwritten tales. ‘In conversation with…’ therefore offers readers a chance to share an abridged version of these conversations*.

*(In view of the need to condense 3 hours of conversation, the original interview text has been abridged, and then reviewed, revised and approved by JSM).*

**“It was a ruddy great earthquake going on! Then it stopped and there was dead silence. And then all of a sudden, CRASH!**

**We’d been having avalanches from this glacier, about one or two a day in the distance, and we had got quite blasé about it. But now they all came down at once.”**

## Q. Sibree is an unusual middle name. Where does that originate from?

It’s a family name—originally a Huguenot name. My great-grandfather was James Sibree, and he was a remarkable chap. He was a master builder up in Hull and ended up in Madagascar building churches. At the time he went, there were very few stone buildings at all in Madagascar; in fact the only stone building was the Royal Palace. So when he got out there in 1863, first of all he had to learn the language, then he had to find the sites. They’d got no tradition of stone masonry and he had to teach them how to dress the stones, quarry the stones and everything else stone related. He also did quite a bit of preaching and Sunday school work and was quite a naturalist; he wrote all about the trees, and the flora, and the fauna and all the rest of it. In time, he came back to England and went to college where he did theological training. Afterwards, he returned to Madagascar as an ordained minister and started the Theological College. In the end, he spent 50 years in Madagascar and wrote widely in Malagasy and English. So yes, he was quite a character.

## Q. I believe your father also worked as a missionary?

Yes he was a medical missionary and my parents ended up in China where I was born. He was a typical missionary doctor; he had to do everything: medicine, surgery, gynaecology, obstetrics, anaesthetics—everything. I was 5 when we left China and I can only vaguely recall scenes by the sea. I remember waking up one morning, looking out from the house, and seeing the rising sun dancing on the water. I remember thinking, ‘We’ve got another day of absolute joy’. It’s one of these things you look back at and think never again would I experience quite that unalloyed joy.

## Q. Do you think it was your father’s medical influence that steered you into a career of medicine?

I suppose so, though because we have lots of doctors in the family, I certainly went through the usual phase of thinking ‘anything but’. I realised, however, that they all seemed to enjoy their lives, and it is silly not to do something simply because everybody expects you to do it. Cutting off your nose to spite your face.

Funnily enough, I nearly went into the jam-making business! When I was 15 going on 16, an old boy of Rydal School came along and asked the headmaster if he could recommend ‘a likely lad to join the family firm’. Well I was duly invited to go and see the factory and decide if this is what I wanted to make a career of. I remember going to Liverpool and being taken round the factory. It was jam making on an industrial scale and I was very impressed. I’m sure, however, it was aimed at the lower end of the market for they bought in whatever was the cheapest fruit, including turnips and any old squash, and then the technicians worked out recipes to turn it into whatever they decided to call it! I remember particularly the raspberry jam, which was made from this kind of basic mush, and for the pips they had got little chips of wood and thrown them in!

## Q. Instead, however, you ended up at Birmingham Medical School. Did you enjoy it there?

Yes I did, and that was a surprise, for at school, whilst I was very happy, I was considered to be quite dim. I was however dyslexic but the term and not yet been invented. Anyway, at Birmingham we had some very good teachers. One particular chap—Arnott, the professor of medicine, was years ahead of his time. I remember him at the beginning of our final year standing up and saying, ‘Now gentlemen, you’re adults and you know what you’re lacking in or what you need. There’ll be no signing in or anything this year. You can come and go as you want. We give you this menu, you choose. You should be able to sort out your own education’. It was great and I feel I owe a lot to Birmingham.

## Q. Whilst there, you developed an interest in flying which led to your later job in the Royal Air Force.

Yes, I joined the air squadron at university. I learned to fly early on in Tiger Moths, and then we changed to Chipmunks. We flew from Castle Bromwich, which has now been developed as an industrial site, but in those days it was a grass airfield. I mean literally just a field with hangars and a control tower but no actual tarmac runways.

In 1956, after I’d done my house jobs, I wanted to travel. In those days travel was, in real terms, far more expensive than it is now, and so the chances of being able to get abroad were pretty small unless you were pretty wealthy. I knew with the RAF I could get posted overseas so I stuck with them. Initially, I was posted to be a pool medical officer to the fighter command in the southern district, UK. This wasn’t ideal so I managed to find the phone number of the chap in the Air Ministry who did the RAF postings, and after about 3 or 4 months I rang him up and, as luck would have it, he offered me Hong Kong.

## Q. Did you enjoy your posting in Hong Kong?

Myself and my newly married wife Betty had a very happy two and a half years there. The commander, Group Captain Smythe, was a climber and an Alpine Club member. He’d led one service expedition to the Himalayas in the days when there were very few expeditions. One day he said, ‘We must organise a mountain rescue team in Hong Kong’.

Somewhat surprised I replied ‘You’re going to have a job to convince the Air Ministry that this is necessary’.

‘Oh,’ he said, ‘you imagine, one of our Vampire pilots has engine failure and has to bail out, and lands on Ma On Shan where he breaks his leg. Who is going to rescue him?’

Well this 3000 ft elevation was heather-covered all the way to the top, but somehow he managed to sell this unlikely story to the Air Ministry and we formed our own mountain rescue team that used to go out rock climbing regularly. As it happens, years later I found a guidebook of rocks in Hong Kong, and there was a Milledge Buttress. I’d quite forgotten that I’d discovered this!

## Q. Following your posting in Hong Kong, you returned to work in Southampton.

Initially I worked at the Royal South Hants, then moved to the Southampton Chest Hospital where I worked under a chap called William Macleod. He was a very impressive clinician and suggested we ought to be doing lung function testing, which was an unknown entity in this country. Now as far as I was concerned, respiratory physiology was one of the dullest parts of the medical curriculum, but he gave me a little green book: Comroe *The Lung*, and when I started reading this book and thinking of applying it to patients who were breathless, the whole subject became alive. So, we set up a little lung function lab with only a recording spirometer to do vital capacity and FEV_1_. It was cutting edge science at the time.

## Q. Whilst in Southampton, you heard about The Silver Hut expedition.

I was reading *The Telegraph* and saw that Sir Edmund Hillary and Griff Pugh were going to take this expedition to study the long-term effects of really high altitude. And I thought to myself, ‘Oh gosh! That would be great’. Well, I found Griff Pugh’s address, wrote to him, and surprisingly, almost by return I got a letter saying that they had already picked their team, but if I happened to be passing, to drop in. Well, I made sure that the very next day I just happened to be passing! The plan was that we would go out in the autumn and set up the high-altitude station. The climbers would then return home, but the scientists would stay over winter, and then in the spring we’d do an 8000 m peak standard, old-fashioned, siege-type expedition. Ed’s thought was that it would be interesting to know whether people who’d spent the whole winter at high altitude would do very much better than people who just came out and did the usual 3 or 4 weeks acclimatisation.

## Q. Nine months in very close proximity to each other must have been hard.

Not at all. I think as far as that’s concerned, we all got on very well. I think one of the secrets was that we were very busy and we all had our jobs. It also helped that at the end of a hard day’s work, we could just down our tools, go outside and jump on the skis. We could get away from everybody, and just ski down this lovely glacier. The problem was we then had to spend a long time trudging back up with the skis on our shoulder.

## Q. The national geographic shows a remarkable photo of your bathing facilities.

Well you have to understand that up there at 6300 m, all the water has to be produced by melting snow, so it is very precious and in short supply. Bathing therefore was pretty scarce. We did, however, do a series of experiments over a period of 2 or 3 days, in which we tonometered blood. Griff had got a big water tank to do this as it had to be done at 37°. Well, when we finished with this water we couldn’t use it for drinking, so I thought, ‘Oh well, this is a chance to have a bath’. So I put it onto the stove, hopped in and had a bath in it. Lahiri took the photo of me in the bath. Yes, it achieved National Geographic status. I must say, I did offer it to other people, but they didn’t take me up on it.

## Q. Unfortunately, you had to descend back to Kathmandu somewhat earlier than planned on the expedition.

At the end of the expedition we had planned to climb Makalu to test the theory about acclimatising over winter. The French had climbed it with oxygen, however, we were planning to do it without. We also undertook a lot of physiology at advanced base camp at 6300 m—ECGs, exercise tests, VO_2_ max, and had planned to do more further up the mountain. By this time Ed had come out; however, he had had to go back and forth to Kathmandu to sort out the political problems arising due to our first ascent of Ama Dablam. He had therefore lost any acclimatisation he had, or much of it, and on his return to Makalu he wasn’t going well. One evening, we were all in the mess tent, just about to have supper, when we heard an odd sort of noise. Myself and Mike Ward went out to find that Ed had had a stroke. He could hardly talk, and his left side was paralysed, facial weakness, the whole bag of tricks. Anyway, I ended up descending with him and what a wretched journey that was. The first thing that happened was that my camera got dust into it somehow and stopped working. The shutter jammed so I couldn’t take any photos. Then, the weather turned pretty nasty and we had an awful lot of rain and mist and so on. And lastly, we were both thoroughly fed up—Ed because of what had happened and what lay ahead in the future: he was still a young man, and I because I’d missed the possibility of going high on Makalu.

## Q. On your return to the UK, you continued working with Griff Pugh?

Well things were so casual in those days, so he managed to get me an MRC grant for 3 months before the expedition and for 4 months after the expedition. I thus spent 4 months clearing up and writing up the expedition. By then, my wife and I had decided that we wanted to work in the third world. I was subsequently introduced to a chap visiting from the Christian Medical College in Vellore: Dr Jacob Chandy was an Indian neuro-surgeon, trained in America, who’d gone back and opened the first neuro-surgical unit in India. Well we met him, sort of ‘under the clock at Victoria Station’ and soon after moved to Vellore…and stayed for 10 years.

## Q. What did your job out there involve?

I was mainly there as a chest physician, but I also did a bit of teaching in physiology. We established a TB clinic, and as there was an established cardio-thoracic surgical unit and I had some experience from Southampton, I joined that team. We were moving from closed to open-heart surgery, and to begin with I was very much part of the pump team. We then opened an ITU and spent quite a lot of time there supervising the ventilation and such sort.

## Q. In this time you also went to work in San Francisco.

Yes. After 5 years we had a year’s break and I felt that what I needed at that stage was proper training in research methods. So I got in touch with John Severinghaus, as he was one of the few people who were interested in high altitude, and he arranged for a research fellowship through the American Thoracic Society to whom I’m very grateful. I spent a very happy 14 months with him looking into the mechanism of acute pulmonary oedema. That was a wonderful time.

## Q. On your return to England, you went to work at Northwick Park, and remained there for the rest of your clinical career.

Well that came about by a chance meeting with John Nunn at a drinks reception. He negotiated a job for me, initially as a full-time MRC employee at Northwick Park working in his division. After 1 year, my job role was split 50 % NHS and 50 % MRC. I worked as a chest physician in ITU and set up a lung function lab. It was delightful and I thoroughly enjoyed clinical work, but by the time I retired in 1995 on my 65th birthday, I was quite ready to leave.

## Q. Throughout your clinical career, you continued your research trips abroad.

Yes I did. Initially there was the Mount Kongur trip with Mike Ward in 1981. It was a wonderful trip. They got permission to climb Kongur, which was then probably one of the highest unclimbed peaks in the world, just short of 8000 m, and Chris Bonington was the climbing leader. Mike asked me to organise the research and I thought that we could study the differences between ourselves, ordinary sort of club climbers and not Olympic-class athletes at all, with the four climbers who were almost Olympic-class mountaineers. The idea was to see whether, if you had been at altitude lots of times previously, did it alter your physiology? One of the studies used an immunofluorescent technique for measuring erythropoietin (EPO), so I looked at EPO levels on ascent. We saw, as other people had found already, that EPO levels go up very quickly and then come down very quickly; however, you still go on producing a lot more red blood cells. That’s still an area that’s not really solved; a paradox in a way. The new thing really was that we could show that after you came down to low altitude, now with an inappropriately high haematocrit, that the erythropoietin levels went below control standard values. An interesting finding.

One particularly nice thing about that trip was that when the climbers were up the mountain, we hadn’t got a lot to do. We were, however, in an area where there were quite a lot of modest 5000 m peaks, so we could go off with 2 or 3 days’ food on our backs and knock off one of these 5000 m peaks. We ended up climbing about five or six of them. Delightful!

## Q. And am I correct in thinking you were the only non-American on the American Medical Research Expedition to Everest (AMREE)?

Yes, and I was very chuffed with that. AMREE was very much the son of the Silver Hut: we did similar studies, plus some others, but with more modern equipment; well, modern for those days. It was extremely successful of course, and as you know, Chris Pizzo got the first alveolar gas samples on the summit. I also celebrated my 51st birthday on it…and my 80th on the Margherita Hut expedition.

## Q. You also undertook a research trip to the Himalayas sponsored by Saga?

Yes. In 1991 I went on an expedition to the Indian Himalayas, to Jhansi. It was a peak that had been climbed from the north, but we were attempting to climb it from the south. It was a bit of an old man’s expedition; we were all over 60 except for the youngster who was 59! We hadn’t got much going for us, but the one thing we did have in abundance was age. So the leader of the expedition, a wonderful chap, got in touch with Saga and managed to get them to sponsor us. A SAGA sponsored trip!

One night, when we were not far off the summit, at three o’clock in the morning, we woke up and all hell had broken loose! We hadn’t got a clue what was going on. The whole tent was moving as if you’re in a rowing boat. It was a ruddy great earthquake going on! Then it stopped and there was dead silence. And then all of a sudden, CRASH! We’d been having avalanches from this glacier, about one or two a day in the distance, and we had got quite blasé about it. But now they all came down at once. Well, the next morning, we thought we would have another crack at this thing but were unsuccessful. But at least we came back with a novel excuse for not climbing a mountain: we were beaten by an earthquake.

## Q. Your involvement in research led to you co-authoring the highly acclaimed *High Altitude Medicine and Physiology* book. How did this come about?

In 1975, Mike Ward had written what was really the first textbook of mountain medicine. I had in fact reviewed it for the *The Lancet.* In 1984, the two of us were on an expedition to Pike’s Peak and I asked if he had thought of doing a second edition of it. As the first edition had not sold very well, probably because at the time very few people were interested in the subject, his publishers were not interested, but they gave him a kind of release which meant that he could cast around and find another publisher—which he did. We were subsequently advised to involve a third author, preferably somebody from the other side of the pond, and John West sprung to mind immediately. Well, even though we thought ‘He’s published umpteen books of his own. He won’t want to join a couple of amateurs like us’, we asked him on the off chance, and to our surprise, he jumped at the idea. Now, it is currently in its fifth edition and that’s been a great stimulus in retirement to keep up with the whole subject.

## Q. Additionally you are still heavily involved in the Diploma in Mountain Medicine, which you helped set up.

The UIAA (Union Internationale des Associations d’Alpinisme) has this medical commission, which at the time was trying to achieve a sort of basic curriculum for numerous altitude medicine courses that had been set up on an individual basis on the continent. The underlying idea was that if someone held the UIAA-recognised diploma, you would know that the person was ‘above board’ and had had the necessary medical training. Myself and David Hillebrand were representing the British Mountaineering Council at the time and were undecided as to whether we should have a diploma course in Britain. Initially, we were of the opinion that ‘climbers don’t like certificates and all this kind of business, so there would be not much call for it’. We then thought of the burgeoning business of commercial expeditions to Everest and other 8000 m peaks. Stories of unscrupulous companies deliberately not having a doctor or any medical kit in their team but rather relying on the fact that there were other expeditions at Everest Base Camp that could look after any of their clients if they got into trouble, were becoming more commonplace, and quite frankly this was pretty off. So we thought that the UIAA Medical Commission should make some kind of a statement saying these expeditions required a medical officer. That then led to the question, ‘Well, OK. If you insist that there be a medical officer, can it be anybody? Can it be a dermatologist or a gynaecologist?’

‘No, it should be someone who knows something about mountain medicine’.

‘How are they going to show that they know something about mountain medicine?’ ‘Well, we’ll have to have a diploma’.

And that is how the diploma started in this country, and the rest, as they say, is history.

## Q. I would like to finish with a few generic questions to which you may, or may not have answers. Do you have a prized paper?

It was probably one of the Sherpa papers written with Lahiri, *Respiratory control in lowlanders and Sherpa highlanders at altitude*

## Q. If I was to give you a fully-funded lab, and all the time in the world, what are the questions you would go to answer now?

Oh gosh! Well perhaps the EPO question as that certainly is a paradox that has not been answered. How it is that when the EPO values have fallen back to values barely above control, the stimulus to producing new red cells, erythrogenesis, continues? One or two hypotheses have been disproved, so the issue now is that you need to work out the hypothesis that you would want to test, and then think of a protocol for the study that would answer it. Additionally, another area of interest, which I initially looked at in the Margherita Hut but never followed up, is the business of the pulmonary hypoxic pressor response and its failure to be reversed upon removing the hypoxia. A fascinating subject.

## Q. And finally, you have obviously worked with many hugely influential characters over the years. Is there anyone who stands out as a key influence?

Gosh, there are so many: Melville Arnott, Bill McLeod, the three Johns—John West, John Severinghaus, and John Nunn. And obviously Griff Pugh. He was a difficult character at times, who didn’t suffer fools gladly, but a wonderful scientist and friend.

